# Next-Generation Sequencing for Clinical Management of Multiple Myeloma: Ready for Prime Time?

**DOI:** 10.3389/fonc.2020.00189

**Published:** 2020-02-25

**Authors:** Niccolo Bolli, Elisa Genuardi, Bachisio Ziccheddu, Marina Martello, Stefania Oliva, Carolina Terragna

**Affiliations:** ^1^Department of Clinical Oncology and Hematology, Fondazione IRCCS Istituto Nazionale dei Tumori, Milan, Italy; ^2^Department of Oncology and Onco-Hematology, University of Milan, Milan, Italy; ^3^Department of Molecular Biotechnologies and Health Sciences, University of Turin, Turin, Italy; ^4^Seràgnoli Institute of Hematology, Bologna University School of Medicine, Bologna, Italy; ^5^Seràgnoli Institute of Hematology, Azienda Ospedaliero-Universitaria Sant'Orsola-Malpighi, Bologna, Italy

**Keywords:** multiple myeloma, next generation sequencing, prognosis, personalized medicine, genomics

## Abstract

Personalized treatment is an attractive strategy that promises increased efficacy with reduced side effects in cancer. The feasibility of such an approach has been greatly boosted by next-generation sequencing (NGS) techniques, which can return detailed information on the genome and on the transcriptome of each patient's tumor, thus highlighting biomarkers of response or druggable targets that may differ from case to case. However, while the number of cancers sequenced is growing exponentially, much fewer cases are amenable to a molecularly-guided treatment outside of clinical trials to date. In multiple myeloma, genomic analysis shows a variety of gene mutations, aneuploidies, segmental copy-number changes, translocations that are extremely heterogeneous, and more numerous than other hematological malignancies. Currently, in routine clinical practice we employ reduced FISH panels that only capture three high-risk features as part of the R-ISS. On the contrary, recent advances have suggested that extending genomic analysis to the full spectrum of recurrent mutations and structural abnormalities in multiple myeloma may have biological and clinical implications. Furthermore, increased efficacy of novel treatments can now produce deeper responses, and standard methods do not have enough sensitivity to stratify patients in complete biochemical remission. Consequently, NGS techniques have been developed to monitor the size of the clone to a sensitivity of up to a cell in a million after treatment. However, even these techniques are not within reach of standard laboratories. In this review we will recapitulate recent advances in multiple myeloma genomics, with special focus on the ones that may have immediate translational impact. We will analyze the benefits and pitfalls of NGS-based diagnostics, highlighting crucial aspects that will need to be taken into account before this can be implemented in most laboratories. We will make the point that a new era in myeloma diagnostics and minimal residual disease monitoring is close and conventional genetic testing will not be able to return the required information. This will mandate that even in routine practice NGS should soon be adopted owing to a higher informative potential with increasing clinical benefits.

## Molecular Pathogenesis of Multiple Myeloma and Related Monoclonal Gammopathies

Multiple myeloma (MM) is a post-germinal center B-cell neoplasm characterized by the accumulation of clonal plasma cells, the production of a monoclonal antibody, and end organ damage (1). MM is preceded by asymptomatic stages of disease in virtually all cases. These are monoclonal gammopathy of unknown significance (MGUS) and smoldering multiple myeloma (SMM). MGUS is a stable condition and progresses at a low rate of 1%/year (2). SMM patients on the contrary have a much higher risk of transforming to MM, but this risk is not constant: it averages 25% per year for the first 2 years, and then declines reaching levels similar to MGUS in patients who did not progress 10 years after diagnosis (3).

The pathogenesis of the disease comes from genomic alterations thought to occur in the germinal center of a secondary follicle of a lymph node. Particularly, cytogenetic changes such as hyperdiploidy or translocations involving recurrent oncogenes and the immunoglobulin heavy chain locus are considered initiating events (4). In some individuals, inherited alleles (i.e., germline polymorphisms) can increase the risk of developing MM but this is considered a rare occurrence and all alleles identified so far have been shown to only confer a small risk (5–14). On the contrary, gene mutations are frequent in newly diagnosed MM (NDMM) and have been particularly characterized since the advent of next generation sequencing (NGS) technologies (15–22). The most commonly involved genes pertain to the MAPK pathway, the NF-kB pathway, the DNA damage response/TP53 pathway. Interestingly, great heterogeneity of the mutational spectrum of NDMM has consistently been reported, such that (i) only few genes are recurrently mutated in a significant fraction of patients, with a high number of genes mutated in <10% of them; (ii) within a single patient, often mutations are only present in a fraction of cells, i.e., they are subclonal (23) or may present in lesions from some anatomical locations but not others (24, 25). Consequently, gene mutations are thought to be late events that contribute to MM heterogeneity and impact disease progression more than its initiation (4). In fact, NGS analysis of sample series has shown variable degrees of spontaneous evolution of genes mutations, cytogenetic lesions and mutational signatures (26–33). This suggests that MM evolves in discrete steps not just clinically but also from a molecular point of view, with the acquisition of subsequent genomic lesions that underlie an increasingly aggressive clinical behavior.

Following examples from other cancers, several efforts have been put in place to use genomics to explain chemoresistance in relapsed disease. Indeed, serial analysis of pre-treatment and relapsed MM samples again showed a tendency toward evolution, where a change in subclonal structure was often observed together with an enrichment of high-risk features (17, 34, 35). Confirming the higher prevalence of high-risk lesions in more aggressive stages, NGS analysis of primary plasma cell leukemia, a rare extramedullary presentation of a clonal plasma cell dyscrasia, showed increased prevalence of *TP53* mutations and del(17p) (36).

Overall, experimental evidence so far suggests that myeloma progression, both spontaneous in asymptomatic stages and at relapse after treatment, is linked to its heterogeneous subclonal composition. Consequently, both the size of the tumor mass and the intrinsic biological features of each subclone must be studied if these biological advances are to be brought to clinical practice to improve prediction of MM evolution.

## Current Clinical Approach to Prognostication in Monoclonal Gammopathies

Recent advances in NGS technologies have provided us with an unprecedented amount of data on the cell-intrinsic features associated with the natural history of the disease. Despite these advances, diagnostic criteria still segregate MGUS from SMM based on surrogate measures of disease burden (i.e., percent plasma cell bone marrow infiltration and serum levels of the monoclonal protein), and SMM from MM based on the presence of end-organ damage or myeloma-defining events (37).

SMM is a clinical diagnosis that encompasses a wide range of cases, from indolent ones that behave similar to MGUS to aggressive ones that are to progress quickly to MM. Consequently, several risk factors have been proposed to stratify patients based on the risk of progression. Some are based on laboratory values, others on imaging, but only few on intrinsic characteristics of tumor cells: among those, high-risk cytogenetic lesions, gene expression profiling and abnormal immunophenotype (3, 38, 39). However, only rarely such complex techniques are performed in routine diagnosis of SMM. Consequently, the most commonly used risk model for SMM progression relies on % bone marrow plasma cells, levels of the monoclonal protein and free light chains (2, 40–43). Unfortunately, different risk scores show poor overlap (44) and imperfect prediction, which is likely due to the fact that direct measures of the clone size and its intrinsic biological features are not captured by the most widely used approaches.

In NDMM, prognosis has historically been dictated by serum levels of albumin and beta-2 microglobulin within the international staging system (ISS) (45). Only recently the ISS has been complemented by LDH levels and FISH analysis of del(17p), t(4;14), t(14;16) in plasma cells to provide a more accurate measure of risk (R-ISS) (46). Additional studies have shown how the addition of further FISH markers, or the use of SNP arrays can refine prognostication (47–50), but novel prediction scores lack prospective validation and wide applicability so far. Therefore, a lot of variability exists regarding which culture conditions and FISH probes should be used to identify different chromosomal abnormalities (51). This variability stems from the standard practice of each center, but also from national and international guidelines which may slightly differ, and from availability of reimbursement. For example, NCCN guidelines version 2.2020 (https://www.nccn.org/professionals/physician_gls/PDF/myeloma.pdf) recommend FISH on plasma cells for del(1p), gain (3 copies) or amplification (>3 copies) of chromosome 1q, del(13q), t(4;14), t(11;14), t(14;16), t(14;20), and del(17p) at time of diagnosis. An alternative staging system to the IMWG R-ISS is the Mayo Clinic mSMART 3.0 (www.msmart.org) that stratifies myeloma patients into high or standard risk groups. The former includes del(17p), t(4;14), t(14;16), t(14;20), amp(1q), high risk gene expression profile signature, high plasma cell S-phase, combinations of 2 or more high-risk genetic abnormalities. The latter includes hyperdiploidy, t(11;14) or t(6;14).

## Unfulfilled Promises in Molecular Diagnosis Applied to Myeloma

NGS has shown how MM genome is characterized by conspicuous heterogeneity and a subclonal structure that gains complexity as the disease evolves. The hypothesis underlying this review is that a precise characterization of this complexity in each patient offers a better possibility to predict, if not prevent, disease evolution and thus improve clinical management. On the contrary, risk scores used in current practice are blind to this complexity, as they rely on clinical and laboratory markers and on a handful of cytogenetic lesions assessed by FISH that are not enough to capture the described complexity and measure MM aggressiveness. NGS thus has the potential to produce a Copernican revolution in how we approach plasma cell dyscrasias in the clinic, i.e., moving from surrogate measures of tumor burden to actual quantification of disease extension coupled with detailed biological analysis of the subclones present in each case.

However, 9 years since the first NGS study in MM has been published (15), clinical practice has been relatively slow in embracing NGS as a diagnostic technique that may complement the standard approach based on morphology, FISH and flow cytometry. Likely, MM intrinsic heterogeneity and the variety of treatment options have hampered the rapid identification of novel prognostic and predictive markers, and there is no consensus so far as to whether, and how, NGS should be used to re-define high-risk disease (52, 53). Furthermore, NGS in MM requires cumbersome sample pre-processing with CD138 cell purification in most cases to obtain meaningful results. As an example, NGS studies in acute myeloid leukemia have gained traction in the clinic in a much quicker way, owing to a lower disease complexity and clearer translational results (54–57).

However, we believe that part of the explanation could also stem from a knowledge gap between routine clinical care and the field of NGS analysis. In fact, NGS can be perceived as a slow, complex, expensive technique that returns results that are hard to interpret and reproduce, and with little clinical value. On the contrary, a targeted NGS panel can inform on gene mutations, aneuploidies, segmental copy-number abnormalities (CNAs), and translocations in a much more comprehensive way than FISH, karyotyping or SNP arrays (58), at a lower cost than a comprehensive FISH panel, with short on-hand processing time and turnaround time and promising clinical correlates. Here, we propose that NGS should be part of the initial diagnostic workup of every NDMM case, at least in tertiary care centers and within clinical trials. This will allow a more precise definition of prognostic and predictive factors that are of clinical significance today. Furthermore, the creation of large NGS data banks that could be mined in the future will allow the quick discovery and validation of novel genomic correlates of prognosis and treatment response that could only become relevant for future treatments.

## Next Generation Sequencing

### The Technique of Next-Generation Sequencing

In the last decade, the introduction and development of new sequencing technologies opened new biologic scenarios especially in onco-hematologic fields (59). Currently, Illumina (San Diego, CA) and Thermo Fisher (Waltham, MA) are the most used platforms. They are referred to as “next-generation,” although effectively they represent a “second generation” of technologies after the irreversible terminator sequencing invented by Sanger. Illumina's integrated NGS instruments use a reversible-terminator based technology. They can read up to 300 bps and importantly, perform paired-end sequencing. This implies that they are able to detect chimeric DNA molecules where the two ends derive from different chromosomes of chromosomal segments, such as in the case of a translocation breakpoint being present in the middle. In fact, many sequencing projects are nowadays aimed at identifing whole-genome translocations and CNAs and not mutations, and to do so a new strategy based on low-coverage, long-insert DNA libraries has been developed to increase the likelihood of identifying chimeric reads at low coverage. This has the potential to represent a new gold standard replacing FISH in the future, since it carries higher accuracy, lower cost and higher throughput. Consequently, Illumina represents the most commercialized NGS platform especially for large genome-wide studies, metagenomics, and gene expression studies (60). Differently, Thermo Fisher sequencing technology, commercialized as Ion Torrent's semiconductor sequencing relies on hydrogen atoms release during DNA polymerization (61). These machines generally provide reads length up to 150–200 bp and are often employed in smaller scale targeted resequencing projects such as those required for diagnostic purposes. As they perform mostly single-end sequencing, their performance in detecting structural variants is weaker.

In the near future, a third generation of sequencing technologies will be widely adopted. The most advanced platforms are provided by Pacific Biosciences and Oxford Nanopores, and they are based on single molecule sequencing without DNA fragmentation, thus producing reads in the thousands of bases. While these are still error-prone machines and not suitable for clinical-grade mutation calls, they may outperform current technologies for detection of structural variants.

Depending on the input DNA and the processivity of the machines, DNA sequencing can be performed at the level of the whole-genome (WGS), the coding genome (whole-exome, WES) or limited areas of interest (targeted panels). These three sequencing strategies have all been variably adopted to investigate MM heterogeneity. Their principal characteristics are resumed in Table 1. Preference between WGS, WES or a targeted panel depends on the type of variants that need to be detected (e.g., mutations vs. aneuploidies vs. structural aberrations) and on the total target footprint. Clearly, smaller footprints allow faster and cheaper analysis, through the possibility of multiplexing more patients into each experiment. Also, IT requirements for downstream analysis are less demanding. For this reason, the choice of the experiment is greatly influenced by the research-diagnostic question, but also by the instrument/technology available and the sample load in each laboratory. Currently, for clinical purposes a targeted panel able to detect mutations, copy number alterations and all the known IGH/IGK/IGL rearrangements represents the most cost-effective solution for risk stratification in MM (21, 35, 58, 62–64). However, this approach is intrinsically limited for research in that it requires prior knowledge of what to look for, and hence it might miss unknown -but relevant- translocations or gene mutations. On the other hand, a WGS approach has the potential to capture the full spectrum of genomic aberrations in MM, but at a much higher cost and time of analysis.

**Table 1:** Types of tests available for genetic/genomic analysis in MM.

	**Target**	**Cost per sample**	**Type of variants detected**	**Number of variants detected**	**Advantages**	**Limitations**
WGS	Whole genome	~€1,500 per sample	Mutations (coding and non-coding), indels, aneuploidies, CNAs, structural rearrangements, signatures	Thousands	Comprehensive genomic characterization	Cost, analysis, storage of data, low depth
WES	Coding genome (2%)	~€500 per sample	Mutations (coding), indels, aneuploidies, CNAs	Hundreds	Lower cost, carries most of clinically useful information, easier analysis	No information on non-coding genome
Targeted	Custom number of genes/regions	Variable	Mutations (coding), indels, aneuploidies, CNAs	Variable	Customizable, lowest cost and complexity of analysis, limited storage required	Not useful for discovery approaches
FISH	Custom number of regions	~€100 per probe	Deletions, gains, translocations	None	Familiar to most laboratories, short turnaround time	No mutations detected, ideal for a low number of probes

### Bio-informatic Analysis

**Figure 1:**
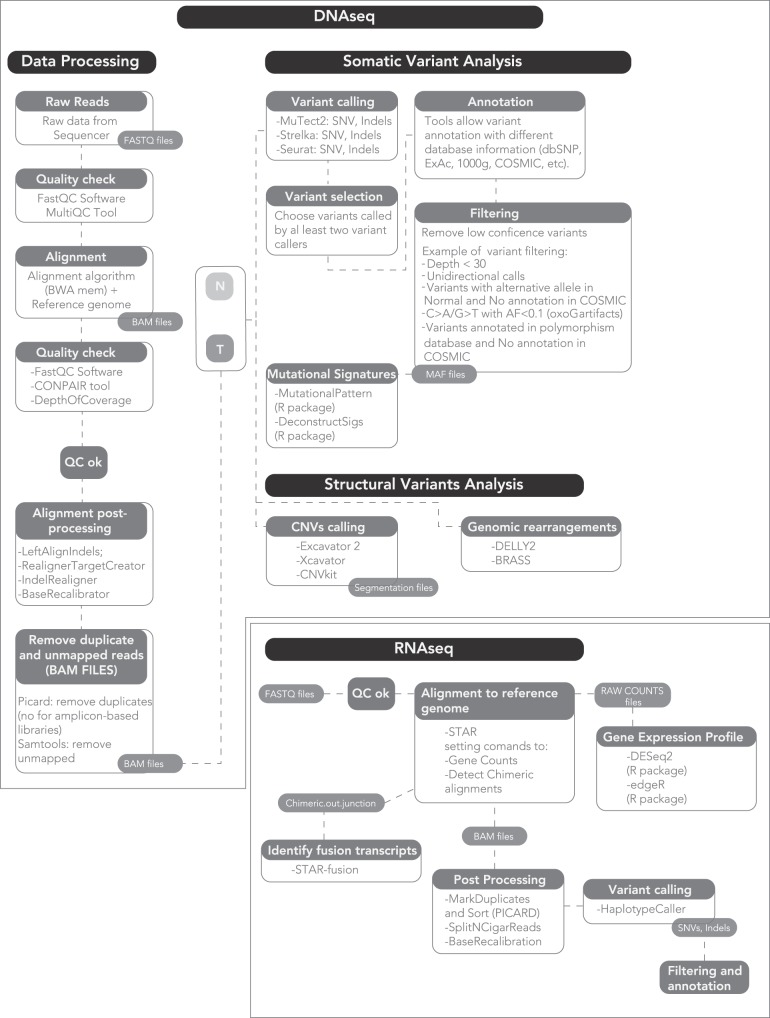
A proposed workflow for comprehensive genomic and transcriptomic analysis.

A standard computer cannot process the output of a NGS machine, nor can a clinical scientist analyze it. Rather, a dedicated machine and a bioinformatics data analyst are required to process the raw sequencing output into data of biological and clinical value (Figure 1). Bioinformatics is the science that combines knowledge derived from biology, computer science and data analysis, with the aim of understanding and giving a role to data from biological processes of a living organism.

The raw output of an NGS sequencer consists of text files, i.e., strings representing the nucleotide sequences and ASCII-coded values that describe their quality. Based on this quality, each read can be included or discarded, or trimmed of the low-quality bases. Subsequently, the strings of disordered sequences present in the raw files are aligned to the reference genome. One of the most widely used algorithms is the Burrows-Wheeler Aligner (BWA) (65). The alignment output is a binary file containing the mapped reads, which can reach hundreds Gb for a single human genome. The aligned files are then processed to analyze multiple information.

To identify single nucleotide variants and small insertions and deletions (SNVs and INDELs), base calls are compared to the reference genome and often to a matched germline sample from the same patient. The latter is required for larger scale discovery effort such as whole-exome or -genome sequencing, where germline individual variation can lead to the inclusion of a large number of false positive somatic calls. Conversely, small targeted gene panels may be analyzed without a germline control, since the sequencing is only performed to identify recurrent oncogenic somatic variants. Importantly, mutation calls are quantitative, i.e., the frequency of the variant (variant allelic frequency, VAF) is calculated and this is proportional to the number of DNA molecules (and thus to the number of cells) bearing that mutation over the total number of sequenced molecules (cells). This implies that the potential to discover a mutation is proportional to the coverage of the sample, i.e., the average number of sequenced DNA molecules per base of the target region. Coverage can be lower for clonal mutations, that are present in all cells of a tumor and thus in 50 or 100% of reads (for heterozygous and homozygous mutations, respectively). However, coverage must be higher for subclonal mutations, i.e., those present in a limited number of tumor cells, and in samples with low purity where a number of DNA molecules comes from contamination, non-tumor cells. Furthermore, coverage is limited by cost: it can be higher if the footprint of the DNA region is little, and must be lower if the whole genome is sequenced. Consequently, there is no set rule to determine the perfect coverage beforehand, and all the factors described above must be considered. As a rule of thumb, a coverage of 30× (i.e., 30 DNA molecules sequenced at each genomic position) is enough to detect clonal mutations and subclonal mutations present in a large fraction of cells, and is typically applied to whole-genome studies. A coverage of 200–500× can allow reliable detection of mutations down to 2–5% of cells and is usually applied to exome studies. A coverage >1000× is usually applied to small targeted panels and can identify variants in <1% of cells, especially with the help of *ad-hoc* algorithms (66). Clearly, this is estimated assuming high tumor purity, which is not always the case.

Different methods to identify variants exist: some are based on allele frequency, counting for each position of the number of normal and alternative alleles, others on probabilistic Bayesian methods, where for each genomic position the probability of observing every possible genotype is returned (67). Importantly, different software can differ in sensitivity and/or specificity, and no gold standard exists to identify variants. However, concordance between different software is usually in excess of 90% for oncogenic variants with high VAF, making calls quite reliable for clinical purposes. However, the concordance can drop to <60% for unknown variants with low VAF, which are nevertheless often discarded in clinic. A new approach that has gained traction is that of using multiple callers to identify variants, and retain only those identified by at least two of them, to increase specificity.

To identify CNAs, NGS offers higher resolution and a more precise identification of breakpoints over conventional arrays, as the depth of coverage in a genomic region is correlated with the total number of DNA molecules sequenced in that region, i.e., its copy number (68). Furthermore, NGS data can be used to evaluate translocations. This analysis is possible when sequencing is performed on a paired-end protocol (i.e., using Illumina machines). Here, opposite ends of the same read that map to distant positions in the same chromosome or different chromosomes are analyzed as they likely highlight a structural rearrangement. Subsequently, single reads spanning the breakpoint can be searched to map the translocation with a base-pair resolution (69).

Another important information that can be evaluated using NGS data is mutational signatures. These are “genomic fingerprints” left around a variant by the biological process that caused it. These are usually processes responsible for DNA duplication and repair, or physical/chemical damage to the DNA. Usually, each process has a preferential activity for a particular nucleotide context, i.e., the base at 5′ and the one at 3′ of the mutation. Combining the six possible types of mutations and the 16 possible contexts, algorithms return the 96-class trinucleotide profile of the mutational spectrum of each sample. This can be further analyzed to extract the mutational signatures (and thus the processes) that contributed to its generation (70).

Last, it must not be forgotten that other types of genetic material can be sequenced. In the case of a cDNA input, NGS can inform on expression levels of genes, expressed mutations, expressed fusion transcripts and splice variants that may have a future in the prognostication in MM. NGS machines can also return information on epigenetic changes related to cancer. Bisulfite-converted DNA can be sequenced to detect methylated cytosines (methyl-seq). Accessible DNA regions can be identified by probing open chromatin with the hyperactive mutant Tn5 Transposase (ATAC-Seq). The activity of transcription factors and the effect of histone modifications can be assessed, along with any other protein-DNA interaction, by sequencing DNA immunoprecipitated with specific antibodies (ChIP-Seq).

## Current NGS Applications in MM in the Clinic

At diagnosis, NGS studies are not routinely performed and FISH is still the main approach to molecular characterization of the cancer cells in MM. This carries the intrinsic limitation of investigating only a handful of CNAs and translocations. However, knowing the complexity of the MM genome the amount of information FISH can return is limited, and so could be its prognostic value in comparison with other approaches.

Conversely, the main current application of NGS in the field of MM relies on detection of measurable residual disease MRD through sequencing of the IGH/IGK/IGL loci. This is a very powerful technique, mostly used within clinical trials to date, and mostly through outsourcing the analysis to a commercial service. In a recent meta-analysis, MRD negativity was found to confer an ~50% relative reduction in the risk of both progression and mortality (71). Historically, molecular MRD has been assessed through a multiplex polymerase chain reaction (PCR) of the IGH locus with consensus primers (72–76) followed by Genescan, heteroduplex analysis, or Sanger sequencing (77). Nevertheless, this approach also amplifies normal B cells resulting in low sensitivity (78, 79). Conversely, an Allele Specific Oligonucleotides (ASO) technique consisting in a real-time PCR with in patient-specific primers and probes has a much higher accuracy and is able to detect up to 1 clonal cell in 100,000 analyzed (80). However, the high rate of somatic hypermutation that occurs in MM cells allows the identification of a molecular marker in only 50–60% of patients. Moreover, detection of the tumor-specific IGH rearrangement often requires cloning of two or more PCR products, resulting in an expensive and labor-intensive procedure. Finally, the ASO qPCR approach does not allow to evaluate the clonal evolution in patients with relapsed MM, thus resulting in false negative results (81). The recent adoption of NGS downstream of consensus primer PCR, resulting in sequencing of all the PCR products, has overcome most of these problems and results in a precise catalog of the IGH, IGK and IGL rearrangements in each case (82). In the diagnostic sample, the tumor-specific rearrangement can usually be easily identified and looked for in the remission sample, always using consensus primers thus increasing the applicability of the technique and allowing a resolution of 1 clonal cell out of a million analyzed cells.

The wide adoption of NGS-mediated MRD measuring with a sensitivity of 10^−6^ is supported by a wealth of clinical data. Recently, Perrot et al. published NGS data from the IFM/DFCI study for young newly diagnosed NDMM patients. In this study, the authors showed that different levels of NGS-MRD cut-off could predict different outcomes in terms of both progression-free (PFS) and overall survival (OS) at both pre- and post-maintenance time points (83). Importantly, the PFS benefit associated with MRD negativity by NGS was similar among the different patient subgroups, thus confirming the theory that MRD is the strongest prognostic marker that overcomes certain adverse risk factors identified at diagnosis (i.e., low-risk cytogenetics and ISS stage II or III), as also reported in other studies (72). The prognostic value of MRD was also independent of previous therapy (transplant vs. no transplant). Moreover, NGS was also explored in other trials for elderly NDMM patients: the ALCYONE and MAIA studies demonstrated that, even if experimental arms (Dara-VMP and Dara-RD, respectively) induced 3- or 4-fold higher rates of MRD negativity compared with control arms (VMP and RD, respectively), the achievement of MRD negativity translated into a significant improvement in PFS independently of previous therapy (84–86). These data are consistent with those of relapsed MM patients enrolled in the CASTOR and POLLUX studies (87–89).

## Novel Insights of Clinical Value Promoted by NGS in Multiple Myeloma

### Smoldering Myeloma

The current clinical approach to SMM is watch-and-wait. However, evidence in favor of early treatment is growing, at least for high-risk stages (28, 90, 91). Therefore, improved prognostic scores that could reliably identify high-risk SMM would address a growing clinical need.

Recently, DNA and RNA-based NGS approaches applied to both individual SMM samples and paired SMM-MM cases have shown that these asymptomatic stages carry a globally lower number of mutations than NDMM (28, 29). However, clonal heterogeneity was observed at this stage as well, implying spontaneous evolution of cancer cells through acquisition of new genetic lesions conferring a proliferative/survival advantage. This was particularly true at the level of single-cell RNA, where some cases labeled as MGUS instead revealed plasma cells with a clearly malignant phenotype (33). Interestingly, analysis of serial samples highlighted two patterns of progression: (i) one where cases evolved from minor or entirely new subclones, often without discernible changes of amount of monoclonal protein, and (ii) another where clinical progression was not associated with genomic changes, and was generally quicker (26, 27, 31, 32). Clearly, the former are true asymptomatic cases that need to acquire new lesions to shift their clinical behavior toward an aggressive phenotype, but in the latter case two scenarios are plausible: these are either indolent cases that evolve due to changes in the microenvironment (92, 93), or more likely actual aggressive myelomas that just need more time to accumulate enough tumor burden and/or end-organ damage to meet clinical criteria for progression. Since the advent of NGS, analysis of rearrangements in SMM has been possible at a whole-genome scale. This has been particularly fruitful in the case of MYC rearrangements, which are frequent in MM but hard to study due to promiscuous partners and distant breakpoints. A recent study has clarified how only IGH-MYC rearrangements confer high-risk of SMM progression, mandating that risk scores are updated to reflect this analysis (94). Also complex rearrangements, a newly discovered phenomenon in MM (23), were equally present in SMM albeit at a lower cancer cell fraction (32). Last, a differential timing of activity of mutational processes was observed in SMM: early mutations, likely from pre-cancer initiation stages, arise from the activity of the DNA deaminase AID or from processes associated with cell aging. Late mutations, i.e., the ones arising at the time of disease progression, are more often caused by a cancer-associated mutational process driven by aberrant activity of the APOBEC family of DNA deaminases (32, 95, 96). Therefore, from a genomic point of view, high-risk SMM cases are most similar to NDMM cases and their identification will help stratification of patients (Table 2).

**Table 2:** Papers showing a prognostic value of extended genotyping in smoldering myeloma.

**Study**	**Method of detection**	**What detected**
Bolli et al. (32)	WGS	APOBEC signature
Maura et al. (23)	WGS	Complex rearrangements
Misund et al. (94)	WGS, targeted	IGH-MYC translocations
Boyle et al. (97) (ASH abstract)	WES, targeted	Mutational burden

Limitations of the described studies are several, ranging from low number of samples, to contamination from normal cells in some cases, to an inevitable bias toward higher-risk SMM cases. However, data are promising enough to believe that NGS will in the future unravel the actual determinants of disease progression in SMM and that will have a profound impact on clinical management of asymptomatic patients.

### Newly Diagnosed Myeloma

At diagnosis, MM is staged in three risk groups based on the R-ISS that relies on surrogates of disease burden (albumin, beta-2 microglobulin, LDH) and FISH for three high-risk cytogenetic features (t4;14), t(14;16), del(17p) (46). Clearly, the extended genotyping ability provided by NGS holds the promise of further refining the prediction of such risk (19, 21, 95, 98), as well as that of identifying novel predictive markers that may guide treatment (99–101). However, the prognostic information at diagnosis in MM has historically been only relevant for the patient in terms of management of expectations, as no risk-adapted treatment in myeloma is available. Instead, the landscape is rapidly changing, and four main aspects are to take into account when thinking of prognostication and treatment of myeloma in the coming years: (i) not all high-risk prognostic factors are captured by the R-ISS (52, 53); (ii) novel treatments may significantly change the catalog of high-risk features; (iii) treatment may become risk-adapted soon; (iv) predictors of response, even if devoid of prognostic value, may soon enter clinical practice.

Regarding the first point, the high-throughput genotyping possibilities offered by NGS, coupled with the availability of large datasets amenable to analysis, have highlighted several risk factors that go beyond the ones described above. Perhaps the most cited ones are those included in the definition of “double hit” MM, where features such as amp(1q) in the context of R-ISS stage III and bi-allelic inactivation of *TP53* by means of mutations of one allele and deletion of the other confer poor prognosis independently of the R-ISS. Importantly, the prognostic value of CNAs in chr1q seems limited to amplification of 4 or more copies of the chromosome, a quantitative result that NGS can capture (98). Other markers have a less clear impact. Among those are del(1p) and del(12p)(48) and a rare state of hypodiploidy/hyperaploidy associated with del(17p) (102–104), which is evident by karyotyping and where NGS could be particularly informative. Furthermore, the recent discovery of the poor prognostic value of immunoglobulin lambda translocations and their lack of response to IMiDs (105) highlights once more the value of an “unbiased,” whole-genome approach in discovery of prognostic markers. In contrast, single gene mutations seem to have very little prognostic value in most cases (17, 21). The one exception is the Myeloma XI UK trial, where patients were treated with IMiDs in first line. In this context, *EGR1* and *IRF4* mutations conferred good prognosis, and *ZFHX4* a bad one (19). However, the study of the whole mutational spectrum of NDMM genomes allowed to draw some correlations between hypermutated samples and worse prognosis (17, 22). This concept can be extended to the analysis of cytogenetic lesions, where several papers have highlighted that prognosis is inversely proportional to their number, often independently of their type (21, 47, 49). Lastly, initial reports on the analysis of mutational signatures to prognosis have highlighted that cases with high contribution from APOBEC have worse prognosis independently from the number of mutations and the cytogenetic subgroup (95, 106). Altogether, data collected in the last years are pointing at a much larger array of lesions that need analyzing to accurately prognosticate NDMM, as it looks like survival is influenced by an increasing genomic complexity more than the presence/absence of a handful of genetic lesions. Unsurprisingly, novel risk scores are emerging that take into account a larger number of lesions to improve prognostication in NDMM (50) (Table 3).

**Table 3:** Papers showing a prognostic value of extended genotyping in newly diagnosed myeloma.

**Study**	**Method of detection**	**What detected**
Palumbo et al. (46)	FISH	t(4:14), t(14;16), del(17p)
Carballo-Zarate et al. (49)	Karyotype, FISH (HDMM patients only)	del(1p), amp(1q), t(11;14), del(13q), del(17p)
Bolli et al. (21)	Targeted	197 different events (mutations, CNAs, translocations)
Maura et al. (95)	WES	Mutational signatures
Walker et al. (22)	WES	Any driver gene mutation
Walker et al. (98)	WES	TP53 mutations, amp(1q), t(4:14), t(14;16), del(17p)
Perrot et al. (50)	FISH, Cytoscan HD arrays	Trisomy 5, Trisomy 21, t(4;14), amp(1q), del(1p32), del(17p)
Barwick et al. (105)	WGS	IGL translocations

In addition to the well-established role of genomic lesions in the onset and development of MM, deregulated epigenetic mechanisms are emerging as important in MM pathogenesis and prognosis. In the past decade, several studies have suggested that epigenetic mechanisms via DNA methylation, histone modifications and non-coding RNA expression are important contributing factors in MM. Their relevance ranges from disease initiation, progression, clonal heterogeneity and response to treatment. All of these post-translational modifications (PTMs) can be tested by next-generation sequencing, focusing on the status of a single gene or small group of genes, potentially revealing their impact on patients' prognosis. For example, in MM global DNA hypomethylation correlates with disease progression (107) and poor prognosis (108). Moreover, DNA methylation has been shown to influence the expression of microRNA genes with tumor suppressor functions (109–111). Deregulation of miRNAs expression and function has been suggested to have a clear impact on tumor initiation, progression and metastasis in cancer including MM (112–114). Global analysis of miRNA expression in MM has also revealed a clinical relevance as the analysis could correlate miRNA expression to disease progression, molecular subtype, survival and response to treatment (115–120). More recently, several whole genome sequencing and gene expression studies have underpinned that histone PTMs can model chromatin structure driving complex regulatory networks (121). However, epigenetic mechanisms are far from reaching enough evidence to be proposed as clinical-grade prognostic markers and further work and technological advances are needed before this can happen.

Whole transcriptome analysis, both by microarrays or RNAseq, can also be used to identify gene expression signatures with prognostic value. The University of Arkansas for Medical Sciences (UAMS) group has proposed some years ago the GEP70 test as a significant predictor of outcome, independent of clinico-pathologic and genetic features (122). More recently, the SKY92 signature has been validated and combined with the International Staging System (ISS) to identify patients with different risk disease with high sensitivity (123). Despite extensive validation and convincing results though, gene-expression based prognostic scores have not gained widespread adoption. Problems are a lack of consensus over which signature should be used, and a laborious and non-standardized sample processing and data analysis.

Therefore, genetic and genomic markers are by far the prognostic markers in MM that are closer to clinical adoption. However, high-risk features are also necessarily defined relative to the treatments available. The one exception is del(17p), that is universally confirmed across age groups and treatment types. Examples of less stable features include the t(4;14), which seems to respond well to first-line bortezomib (99), similar to cases with deletion of the TRAF3 gene (124). On the contrary, the negative prognostic effect of *ZFHX4* mutations seems limited to patients receiving IMiDs as first line, as discussed above. Likely, with an increasing array of anti-myeloma agents, this list is going to expand realizing the much-valued paradigm of precision medicine through the identification of further correlates of drug response. Again, this will mandate that extended genotyping is performed at diagnosis for every patient.

Finally, the treatment landscape of NDMM is rapidly changing thanks to the introduction of novel agents and combinations. Risk-adapted treatment is already proposed by some groups, e.g., with respect to performing or not autologous stem cell transplantation (ASCT) in first line for standard-risk patients (125, 126). Conversely, tandem ASCT has shown improved survival in patients with high-risk features and is widely used in Europe in this setting (127). Last, the introduction of minimal residual disease (MRD) monitoring in people achieving deep responses also carries big promises. MRD-negative status seems to predict longer-term survival regardless of the treatment administered and of the risk at diagnosis (83), so that in future clinical trials it may become a new standard endpoint. In fact, many upcoming clinical trials are designed with different treatment arms based on the risk score at diagnosis and on the achievement of MRD-negative status, so that the future may bring innovative strategies to personalize treatment in MM.

### Relapsed and Refractory Myeloma

Much less is known about the genomics of relapsed-refractory myeloma. Initial studies suggest that cases retain a significant heterogeneity, with subclones showing expansion or reduction based on the type of treatment, increased number of mutations, copy-number abnormalities, complex rearrangements and contribution from novel mutational signatures (17, 23, 34, 35, 96). Targeted sequencing studies have highlighted increasing prevalence of mutations conferring resistance to IMiDs (particularly in *CRBN, IKZF1, IKZF3*) and PIs (*PSMB5, PSMB8, PSMB9, PSMD1, and PSMG2*) (62, 128). However, mutated cases are still a great minority and mutations are often subclonal, suggesting that while functionally relevant, the clinical impact of these mutations and their utility to guide further treatment will need validation. To little surprise, single-agent targeting of actionable gene mutations has revealed unsatisfactory in MM so far (24, 129, 130).

On the contrary, analysis of cases refractory to both Pis and IMiDs has highlighted a higher prevalence of high-risk cytogenetic features such as amp(1q) and del(17p) that may explain chemoresistance much more readily (131). The evidence that new mutations and cytogenetic lesions can be acquired at relapse suggests the utility to repeat genotyping at this stage. However, future studies will also be required to assess whether the predictive and/or prognostic value of genomic alterations described in NDMM is conserved in advanced stages.

## Future Clinical Applications of NGS in the Clinic

**Figure 2:**
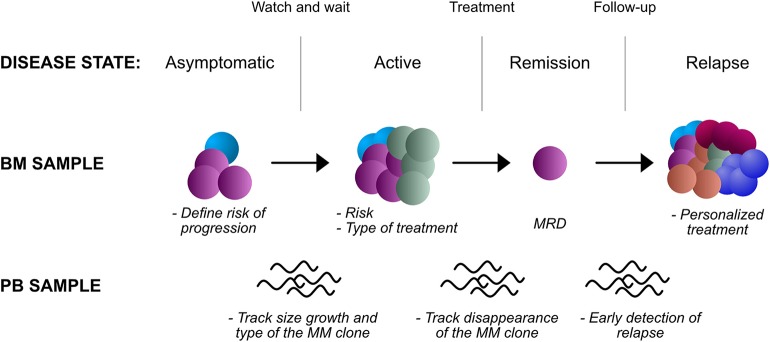
Potential applications of NGS in the clinic, in different disease states and biological samples. BM, bone marrow; PB, peripheral blood; MRD, minimal residual disease.

The recent advances described above suggest that, in the near future, routine management of MM will require such a vast array of genetic findings that an NGS platform would be perfectly suited to address this need (Figure 2). NGS has been already shown to perform as well as or even outperform FISH for structural changes (58, 63, 64). However, cost, turnaround time and regulatory aspects also need to be taken into account. Given the growing number of FISH probes required for a comprehensive characterization of MM and the decreasing cost of sequencing, there is little doubt that soon targeted NGS panels, or even WGS will become cheaper than running, for example, 8 FISH probes as per NCCN guidelines. Turnaround time of FISH can be as short as 24 h. NGS requires slightly more time than this, in the order of 2–3 working days as a minimum. However, treatment of MM is rarely an emergency, and even most acute cases of cord compression or renal failure can be managed with a short course of steroids while waiting for test results. Regulatory aspects are more difficult to discuss as they are also crucially variable from country to country, or even in different regions of the same country. But given the above considerations and the added value of NGS sequencing over FISH, there is little doubt that authorities will allow reimbursement of a cheaper, solid and more comprehensive test than FISH.

### Prognosis in Asymptomatic Stages

In SMM, NGS approaches could capture complex genomic information that may be relevant to stratify risk of progression. Data such as the total mutational burden, the presence of complex rearrangements and IGH-MYC rearrangements in particular, the contribution of APOBEC mutational signature would need a comprehensive assessment through whole-genome sequencing. Techniques such as mate pair sequencing (63) may be particularly well-suited to identify IGH-MYC translocations and complex rearrangements due to their erratic breakpoints and promiscuous partners, features that limit FISH accuracy in detecting these events (132). While costs may limit this approach, a targeted panel of gene mutations, recurrent translocations, and CNAs could still improve our prognostication abilities over current biochemical and imaging techniques.

### Prognosis at Diagnosis and After Induction

In NDMM, NGS analysis will inform on current higher risk cytogenetic markers as per IMWG recommendations. But more importantly, genome-wide copy-number analysis will inform on genome-wide CNAs, aneuploidies and translocations that may change the prognosis of the patient. The simultaneous analysis of gene mutations will provide an added value for prognostication, particularly for *TP53* mutations. Clearly, the quantitative nature of NGS data will also provide an estimate of the number of cells affected by each lesion, which has clinical correlates. Also, a comprehensive mutational analysis of each case will highlight cases that are hypermutated and those with an increased contribution from APOBEC, again markers of high-risk disease.

After induction treatment, NGS can be used to assess MRD as described above. This is probably the most mature application of NGS, and the one that is to see adoption in routine clinical practice first. Hurdles that will need overcoming are the standardization of a protocol to ensure inter-laboratory comparability, access to sequencing facilities and high costs. However, even the more standardized and culturally more accessible use of flow cytometry, despite providing results of similar prognostic value (133), has not gained universal adoption. In the near future, the MRD status may not only be used to inform the patient on his prognosis, but also to guide post-remission treatment -which drugs and for how long- and the need for an autologous stem cell transplant.

### Prognosis at Relapse: Time for Personalized Care?

In RRMM, further BM sampling and genomic analysis may provide improved prognostication and correlates of drug response (Table 4). Examples impacting current clinical practice include the use of second-line ixazomib, that in Italy is only reimbursed upon demonstration of a high-risk cytogenetic status that may be absent at diagnosis. More broadly, recent results suggest that a “targeted treatment” could be closer than expected in the RRMM setting. The most promising results come from venetoclax, a novel inhibitor of the anti-apoptotic BCL-2 protein, which carry single agent activity in RRMM (100) and has shown impressive survival data in combination with bortezomib and dexamethasone. Intriguingly, these advantages are evident in the t(11;14) cytogenetic subgroup (136), again mandating detailed genotyping of the disease. However, BCL2/MCL1 and BCL2/BCL-XL RNA ratio appear to be equally good predictive markers for venetoclax response (134, 137), while *BCL2* mutations and *MCL1* amplification predict resistance (135). This suggests that RNAseq analysis along with DNA sequencing could improve stratification of patients. Use of BRAF inhibitors in *BRAF*^*V600E*^ mutated cases is an approach that failed to fulfill initial promises due to the quick onset of resistance. However, selection of cases where the mutation is clonal, and the combined use of MEK inhibitors could hold promise for the future (24, 130). A more speculative example would be represented by the inhibition of EZH2 in UTX-deleted cases (138). Other mutations could carry a negative predictive value: hotspot *CRBN* mutations suggesting resistance to lenalidomide (62); proteasomal subunit mutations predicting resistance to bortezomib (128); *XPO1* mutations predicting resistance to selinexor (139). Interestingly, most such lesions are often subclonal, and single cell sequencing techniques are starting to uncover an unexpectedly complex spectrum of phenotypes within the myeloma bulk (135). However, despite their potential, such approaches are still far from a possible clinical application.

**Table 4:** Genomic and trasncriptomic correlates of drug response in RRMM.

**Study**	**Method of detection**	**What detected**
Andrulis et al. (129)	Immunohistochemistry	*BRAF*^V600E^
Heuck et al. (130)	Targeted, gene expression profile	KRAS, NRAS, BRAF mutations
Kortüm et al. (62)	Targeted	*CRBN* and CRBN pathway genes
Barrio et al. (128)	Targeted, WES	Proteasomal subunit genes
Kumar et al. (100), Matulis et al. (134), Neri et al. (135) (ASH abstract)	FISH, functional studies, gene expression arrays, single cell RNAseq	t(11;14), BCL2/MCL1, and BCL2/BCL-XL RNA ratio, mitochondrial priming

Finally, novel studies are aimed at incorporating a precision medicine approach in MM. The MyDRUG study (clinicaltrials.gov ID NCT03732703) has six arms where a backbone of ixazomib, pomalidomide, and dexamethasone is used in conjunction with a targeted agent aimed at mutations of any of the following genes: *CDKN2C, FGFR3, KRAS, NRAS, BRAF*^*V600E*^*, IDH2*, or t(11,14). Clearly, inclusion in the study mandates comprehensive NGS analysis.

### Mini-Invasive Approaches for Genotyping and Prognostication

The analysis described above rely on cellular DNA from BM CD138^+^ cells. However, a major breakthrough may be represented by mini-invasive approaches based on circulating tumor cells (CTCs) and circulating cell-free DNA (cfDNA). At diagnosis, these approaches have proven reliable in describing the main clonal gene lesions and aneuploidies in the majority of NDMM patients, where enough tumor cfDNA and/or CTCs are present (140–142). However, a fascinating possibility is that of applying these “liquid biopsy” approaches to add further accuracy to progression free survival (PFS) prediction by defining MRD negativity. In fact, while cellular (e.g., flow cytometry) and molecular (NGS of IgH rearrangements) methods for MRD detection are very sensitive, they are restricted to a single-site BM biopsy, which is in contrast to the patchy and heterogeneous pattern of bone marrow infiltration observed in MM. This may lead to some degree of uncertainty in MRD-negative results, where the disease can still be present away from the bone marrow sampling site. The proof of concept of this caveat is illustrated by the presence of MRD negative patients that still display a monoclonal protein at serum protein electrophoresis (82), and by the poorer survival of a small fraction of MRD-negative, PET-positive patients (143). Some research groups have explored the feasibility of cfDNA analysis to monitor IGH rearrangements by adopting different NGS MRD approaches (144–146). However, the number of CTCs and genome equivalents in cfDNA is so low in MRD settings that these techniques, although feasible, still lack several logarithms of sensitivity before they can reach 10^−6^ and be proposed as standard approaches. Likely, a technical advance is required before the number of tumor genomes sequenced is maximized and this technique provides increased sensitivity.

Another way to longitudinally monitor MM patients and the competitive emergence of subclones through cfDNA is the evaluation of the allelic fraction of known mutations. Some studies have reported the possibility to monitor a single mutation (such as *BRAF*^*V600E*^, *NRAS*^*Q61K*^) by serial sampling of ctDNA (147, 148). This represents a valid approach that takes into account the spatial heterogeneity of MM, and its usefulness would be maximal if the mutation in study is druggable or predicts response to treatment. Another study employed a targeted panel to characterize paired BM and PB samples before treatment. Patients with a higher number of mutations or a higher mutational fractional abundance in PB had significantly shorter overall survival (OS). Moreover, a decrease in ctDNA levels at day 5 of cycle 1 of treatment (C1D5) correlated with superior progression-free survival (PFS) (*p* = 0.017) (149).

Interestingly, ctDNA can be also used to track disease load and clonal evolution of MM by low pass WGS (142). This method allows the identification of copy number alterations even when the tumor load is relatively low. In a pilot study, the potential of cfDNA as a longitudinal marker for disease progression and therapy response has been explored. A patient was monitored before and after therapy both in BM and ctDNA, and efficacy of therapy was evident by decreasing levels of serum free light-chains (sFLC) and concordant trajectory of tumor fraction in cfDNA. The cfDNA copy number profiles on day 0 and day 19 (with no change in management) were concordant. Tumor fraction became undetectable with response to treatment (days 41, 69). However, with relapse extensive clonal evolution occurred (day 224, after relapse) as drug resistance developed. Importantly, the copy number profile of cfDNA and BM on day 224 were concordant. These promising evidences need to be further confirmed by additional studies, and probably pave the way to the use of ctDNA for disease monitoring in the near future.

Once validated, the usefulness of ctDNA, both by IGH rearrangements and/or mutations and/or tumor fraction, together with indirect immunobiochemical markers (e.g., monoclonal protein) and imaging techniques (such as PET-CT or WB-MRI) could possibly help to re-define more precisely the minimal residual disease in MM. Even more interesting, these approaches could be applied to the setting of SMM monitoring, where increase rather than decrease of tumor cfDNA would be observed in progressive cases: this could in theory be less technically challenging and provide earlier detection of symptomatic evolution.

## Author Contributions

NB and CT conceived the idea, reviewed literature, and wrote the paper. EG, BZ, MM, and SO reviewed literature and wrote the paper.

### Conflict of Interest

NB received honoraria from Celgene and Janssen. SO received honoraria from Celgene, Janssen, Amgen and Takeda. The reamining authors declare that the research was conducted in the absence of any commercial or financial relationships that could be construed as a potential conflict of interest. The reviewer RS declared a past co-authorship with one of the authors NB to the handling Editor.
